# Learning regions of interest from low level maps in virtual microscopy

**DOI:** 10.1186/1746-1596-6-S1-S22

**Published:** 2011-03-30

**Authors:** David Romo, Eduardo Romero, Fabio González

**Affiliations:** 1 Bioingenium Research Group, Universidad Nacional de Colombia,Bogotá, Colombia

## Abstract

Virtual microscopy can improve the workflow of modern pathology laboratories, a goal limited by the large size of the virtual slides (VS). Lately, determination of the Regions of Interest has shown to be useful in navigation and compression tasks. This work presents a novel method for establishing RoIs in VS, based on a relevance score calculated from example images selected by pathologist. The process starts by splitting the Virtual Slide (VS) into a grid of blocks, each represented by a set of low level features which aim to capture the very basic visual properties, namely, color, intensity, orientation and texture. The expert selects then two blocks i.e. A typical relevant (irrelevant) instance. Different similarity (disimilarity) maps are then constructed, using these positive (negative) examples. The obtained maps are then integrated by a normalization process that promotes maps with a similarity global maxima that largely exceeds the average local maxima. Each image region is thus entailed with an associated score, established by the number of closest positive (negative) blocks, whereby any block has also an associated score. Evaluation was carried out using 8 VS from different tissues, upon which a group of three pathologists had navigated. Precision-recall measurements were calculated at each step of any actual navigation, obtaining an average precision of 55% and a recall of about 38% when using the available set of navigations.

## Background

Digital technology trends have changed our modern vision about storing, transmitting or visualizing histopatological specimens, under client-server architectures and regular communication networks. Emulation of an actual optical microscope, in virtual environments, is a field currently known as virtual microscopy, a new domain that has generated great expectancies about the role these technologies may play on medical diagnosis, teaching, training, research [1] and evaluation of the pathology laboratory workflow chain [2]. Nevertheless, the actual use of these technologies in clinical scenarios as routine tools still remains limited, among others because of the slowness of the histological digitization, the lack of standard acquisition processes, the long latency times when accessing remote computational systems and the large requirements, concerning computational resources [3]. Development of optimal strategies to cope with these restrictions is then a priority for the field to reach a certain level of maturity. The main impact in terms of a seamless navigation is introduced by the large size of these images. In other words, these systems require huge storing spaces and dedicated communication lines, both increasing the cost of this technology. Smart compression turns out to be a pre-condition for these images to be useful but also acceleration interaction methods need to be devised. Interaction with these data could be speeded up by designing problem-related strategies such as prefetching the required data to the client side before he/she ask it. Likewise, navigation may be highly improved by storing part of this information in adapted cache spaces . Furthermore, most network and computational bottlenecks could be highly improved if Regions of Interest (RoIs) may be set beforehand. However, manual selection of RoIs in images of such size is an impossible task in clinical routine so that automation of this process results an important pre-condition for these systems to be useful, basically because this RoI setting results in probabilistic maps that may be used as initial condition of any pre-fetch or caché strategy.

A classical approach for finding RoIs in natural images has consisted in identifying regions of the image with high spatial edge density [4]. This concept could notwithstanding hardly be applied to histopathological images because they contain regions with high edge concentration without clinical meaning [5]. In medical images, the selection of RoIs has been approached using several methods. For instance, Karras et al. [6], in gray scale images from abdominal cancer, hypothesizes that regions with high density of repetitive patterns were of diagnostic interest. These features were the input to a fuzzy c-means clustering algorithm that classified regions as important or non-important. This technique is not, very likely, applicable to histopathology images because information coming from color, intensity or spatial correlation [7,8] results crucial for identifying diagnostic areas.

In the histopathological domain, specifically automatic cancer diagnosis, [9], the disease was characterized at two levels: cellular, focusing on cell abnormalities, [10,11] and tissular, describing changes in cell distributions [12]. In both cases, this description was performed by low level image characterization and statistical analysis to discriminate normal from cancerous tissues. Oger et al [13] have proposed an automated method for finding RoIs in breast tumor section. The performs an spectral analysis using a rectangular grid which represents the image as a graph, where every node corresponds to a block and every edge is weighted by a 'similarity' between the nodes(blocks) that are connected. A random walk on the data is set by the probability to pass from one node to another. The second and third eigenvectors of the transition node matrix, allow to automatically sort out the blocks by classes. Segmentation of colon glands has been achieved using graphs [14]: a set of primitives are used to segment glands, making use of the object distribution, quantified as the definition of object-graphs.

A pathological diagnosis is the result of a complex series of activities mastered by the pathologist. Classical psycho physical theories suggest that complex visual tasks, such as histological examination, involve high degrees of visual attention [15]. There exists evidence showing that visual systems integrate the constituting low level features of an object [16]. These findings have inspired several computational algorithms that somehow search to capture the main meanign of the low level features [17]. One of the most influential is the one proposed by Itti et al. [18], a pure bottom-up attention model that locates relevant foci, based on a conjoint map of three low level characteristics, i.e., color, intensity and orientation. However, histopathology identification of diagnostic areas (regions of interest) require the association of complex visual patterns in tissues with pathologies or organs [19], through an active search of specific features. This requirement limits the performance of Itti Model for the detection of relevat regions on histopathologic images. An automated method [20] for finding diagnostic regions-of-interest (RoIs) in histopathological images used a modified version of the Itti’s model (Adding entropy as a key feature) to partially establish which areas could be relevant.

In this work we present a novel semi-automatic method that is able to find RoIs from histopathological images. The strategy starts by splitting the VS into an arbitrary partition of subblocks, upon which a distance to a typical relevant subblock, selected by an expert pathologist, is assigned. The metrics is defined as a non linear combination of the projection of each of these subblocks into several subspaces, each defined by different low level features. Finally, a ranking score allows to define several levels of relevancy or level sets of relevance, not necessarily connex. This article is organized as follows: next section will describe the computational method used for the extraction of regions of interest on histopathological images is also described. Section results presents the experimental evaluation, while the method performance to find RoIs is demonstrated by using the precision and recall measures. In the discusion section, we present an analysis of the results and the potential impact of them onto the virtual microscopy field.

## Materials and methods

### Virtual slides acquisition

A total of seven histologic slides were digitized and eight high resolution images (WVS) were assembled using an acquisition system which consisted of a Sony high resolution digital video camera Handycam DCR-HC85 (640 x 480 pixels) coupled to a Carl Zeiss Axiostar Plus microscope, provided with Carl Zeiss 426126 and 456006 adapters (Carl Zeiss, Light Microscopy, Gottingen, Germany). Biological samples were a normal inmunostained pancreas with a captured grid of 64 × 64 microscopical fields (752 × 560 pixels) representing an effective area of 13.114 × 9.641 mm, a neuroendocrine thyroid tumor stained with Hematoxylin-Eosin (160x159 microscopical fields), an atypical thyrod adenoma marked with thyroid peroxidase (91x123 microscopical fields) and four additional specimen stained with Hematoxylin-Eosin, with diagnosis of apendicitis, reactive follicular hyperplasia of a lymph node, leiomioma and normal thyroid, respectively. Virtual slides were stitched using automatic registration with cross correlation as the similarity measure [21] and were stored in JPEG2000 format for latter access and navigation.

### Navigation patterns

**Figure 1:**
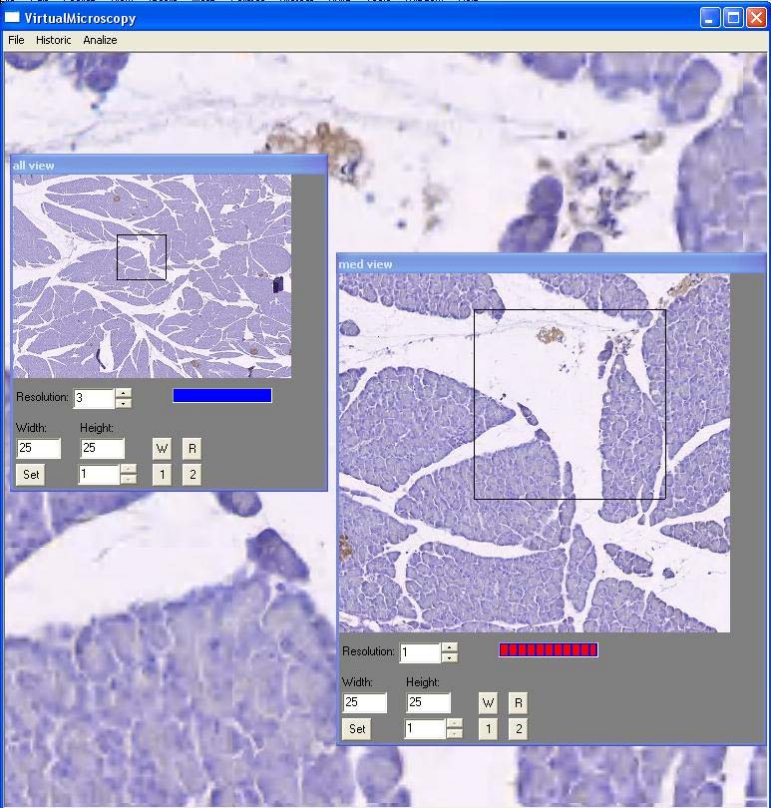
Graphical User Interface of the virtual microscope prototype. This illustration shows a small and medium microscope magnification for lower resolutions while a high enlargement is displayed in the large window. Panning is allowed only in the smaller windows.

Three expert pathologists, each certified with at least five years of experience, were previously trained in the use of a virtual microscope prototype. The Graphics User Interfase (GUI) of the virtual microscope is composed of three windows: the main, the intermediate and the largest magnifications. The former displays the smallest version of the WVS, the thumbnail image, upon which a rectangular re-sizable Window of Interest (WoI) allows a desired selection to be displayed as the intermediate magnification. Likewise, a second re-sizable WoI is drawn onto the intermediate WoI and projected as a new window with the largest magnification. Displacements of a particular WoI are only allowed for the thumbnail (the intermediate WoI moves here) and intermediate (the largest WoI moves here) magnification windows through drag and drop operations. The GUI used in this work is depicted in figure 1.

The virtual slides were randomly displayed to each pathologist. Pathologists were asked to examine the virtual slide until a diagnosis was reached, either the organ recognition, the pathology, or both, using a regular computer screen. During navigation, each WoI request, regarding position, size, resolution and time, was recorded for later analysis. The images used for the method training, correspond to medium magnification versions of the actual virtual slides.

### Proposed method

**Figure 2:**
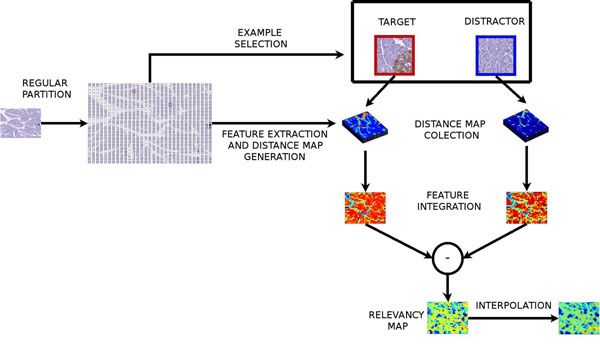
Proposed Method Diagram

The approach herein developed aims to detect RoIs by calculating a local relevance from positive (target) and negative (distractor) examples. The method is illustrated in figure 2

The whole strategy starts by splitting the VS into an homogeneous grid of blocks. In the present work the block size was typically of 70 × 70 pixels, which corresponds to an area of X um2. At this stage, every block is projected to the space of visual characteristics i.e. a block is represented as features in four different spaces, namely color, intensity, orientation and texture. Once the partition is defined, the pathologist selects a typical target (distractor) example that will be used in the learning phase, which is herein understood as the boundary definition of the classification problem, using the typical examples. For doing so, a similarity metrics allows to find the cluster of the target (distractor) examples in the feature spaces. The boundary between these positive and negative clusters is learned from actual navigations.

A similarity map is generated in each of the subspaces for the target (distractor) class and non linearly integrated into a single probability map. The whole process is further detailed hereafter:

#### Feature extraction

The feature choice has been motivated by aiming at having a very general representation of the selected spaces, that is to say, no particular effort was devoted to using characteristics that could describe the set of VS used in the present investigation. The following low-level features has been selected to represent these histopathological images.

• Gray scale and color histogram: This histogram stores information about intensity and color image distribution. For gray-scale images, a 256 bins histogram was used while in For color images, the RGB space was partitioned in 8 × 8 × 8 = 512 bins.

• Local Binary Partition histogram: This texture related feature is obtained as follows, the intensity value of a pixel P is compared with its 8 neighborhood. If the neighbor intensity is larger (smaller), its position is filled with 1 (0). This binary string with eight bins can take values up to 256 and the histogram with 256 values was calculated.

• Tamura texture histogram: among the 6 different Tamura features: coarseness, contrast, directionality, line-likeness, regularity, and roughness, the first three were used since they are strongly correlated with human perception. The space generated by these three features is partitioned in 8 × 8 × 8 = 512 bins and the associated histogram is calculated.

• Sobel histogram: the Sobel operator is one of the most known image processing operators for edge detection. A 3 × 3 operator was herein used and the associated histogram with 512 bins is calculated.

#### Metrics

Provided that every low-level feature was herein represented with histograms, the respective metrics should take this into consideration for evaluating differences. The used metrics were:

• Euclidian Distance

• Histogram Intersection

• Jensen Shannon Divergence

• Relative Bin Deviation

• Relative Histogram Deviation

#### Map integration

The image provides then the geometrical reference frame, upon which different features define several maps, obtained from the different representation and metrics. A particular feature map highlights the relevance of a unique characteristic. Five different features, corresponding to visual perceptions, and five metrics have been herein used to characterize the VS contents. Taking into account that the generated maps are extracted from different features, and that their distance is evaluated using different metrics, each of the maps will have different ranges. Once the set of maps is generated, information was integrated using a non-linear operator, as described by Itti [22]. This operator globally promotes those features maps in which a small number of strong activity peaks, as follows

• Provided that the example block is part of the VS, it is important to devise a mechanism which avoids an excessive promotion of this particular block. This is achieved by reassigning the least distance map value (distance is 0), which always corresponds to the target block, to the second minimum value.

• Map normalization within the interval [0 N], where 0 stands for the maximum distance and N is the minimum so that a similarity function is defined from the distance maps. Our interest is to assign a larger score to the relevance zones.

• The local maximum in a 3x3 neighbourhood and the global maximum are calculated for each similarity map and then the local maximum average is computed.

• Each element of these maps is weighted by (M-m)^2^ so that maps with a global maximum are better scaled than other for which this distance is smaller.

• Finally the set of maps is integrated by adding them out.

After the process of integration is achieved, we have one map representing the target query, and the other the distractor. The final similarity is obtained from the difference between these two maps, as

where N denotes the total number of maps, a_m_ and b_m_ are weight factors in the normalization process and T_m_ (target) and D_m_ (distractor) stand for the normalized similarity and dissimilarity maps, respectively.

This map allows the selection of RoIs when setting a particular threshold for this ranking map, for example with the 10% of the largest ranked blocks.

### Experimental evaluation

The evaluation was addressed to measure the correlation between blocks with high relevance score and the blocks belonging to an observation path. The observation path blocks are marked as I, when the number of visits is larger than 50 % of the visits received by the more visited block, while the estimated ones are marked as E. The conducted experiment consisted then in assigning a binary level of relevance to the observation path blocks and measuring its coincidence level with the largest estimated relevance blocks i.e. blocks whose estimated level of relevance was larger than a 90 %.

The method was in addition compared with other two methods, the Itti’s model and a random block selection. The Itti’s model required an additional step since the saliency map was averaged within the block.

Itti’s model implementation is described in [23].

Precision, the percentage of estimated blocks (E) identified as relevant (I), and recall, the percentage of blocks (I) correctly estimated (E), were herein used to measure the performance as follows:

## Results

**Figure 3:**
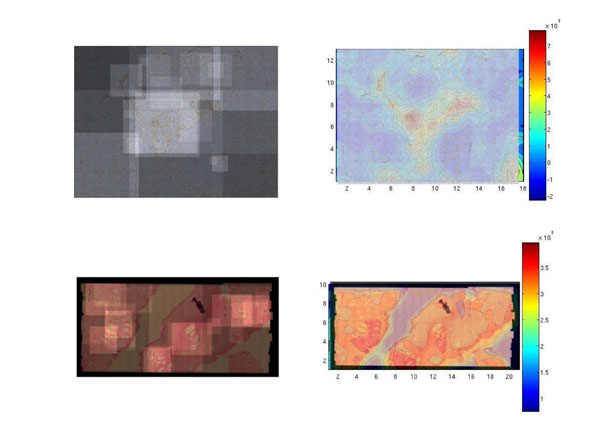
Left: Histopathological image. Visited regions by pathologist are represented by higher intensity areas. Right: Relevancy map obtained with the method.

During the experiment we used the eight virtual slides described above. At the upper and bottom left panels of Figure 3 it is observed a low resolution version of a the VS, of a inmunostained pancreas and a prostate stained with haematoxylin-Eosin. Upon these images the observation paths have been superimposed, in lighter color when more than one pathologist has run over it. The observed result shows the image areas more visited in both cases. The right panels, upper and bottom, show a scale with the results of the proposed method. The scale show in red the more relevant areas and in blue the less ones. Coarsely, these results shows that relevance coincides with what the zones visited by the pathologists in both cases, even when one considers that the two types of navigation were completely different.

Inmunostained pancreas had nearly very few things to explore and pathologists dedicated their time to wander around the image center, basically because Langerhans Islets were located there. However, our method nicely followed this pattern. The prostate sample shows a more distributted pattern which is also followed by our method.

In Table 1. Precision and recall results for each of the methods: the proposed model, Itti’s model and random selection. Both measures were higher in the proposed method. In the case of the Itti’s model, the performance varies according to the particular VS but in any case, larger than the random model.

Overall, precision and recall are consistently higher for the proposed strategy with an average precision of 0,55 and an average recall of 0,38, figures considered as adequate for the retrieval community. In image 3 precision was 1, indicating that every block estimated by our method is relevant, however not every block, marked as interesting, was correctly estimated, whereby the recall was in this case of 0,26. This is mainly due to the fact that the quantity of blocks defined as relevant exceeds the quantity of estimated blocks whose relevancy is larger than a 90 %. An important factor which should be considered in this case is that the RoIs requestes by the pathologists contained the RoI plus regions with no interest which are part of the RoI neighbourhood. Finally, yet the result of the Itti’s model was always poorer than the presented method, it is always better than a simple random selection.

**Table 1:** Precision and Recall results

	Proposed		Itti Model		Random	
Image	Precision	Recall	Precision	Recall	Precision	Recall

1	0,12	0,23	0,01	0,03	0,05	0,09
2	0,43	0,16	0,3	0,11	0,25	0,09
3	1	0,26	0,09	0,02	0,35	0,09
4	0,57	0,67	0,14	0,17	0,08	0,08
5	0,57	0,2	0,1	0,03	0,28	0,1
6	0,48	0,73	0,17	0,27	0,06	0,09
7	0,5	0,42	0,31	0,26	0,11	0,09
8	0,69	0,32	0,05	0,08	0,21	0,1
Average	0,55	0,38	0,14	0,12	0,17	0,09

**Figure 4:**
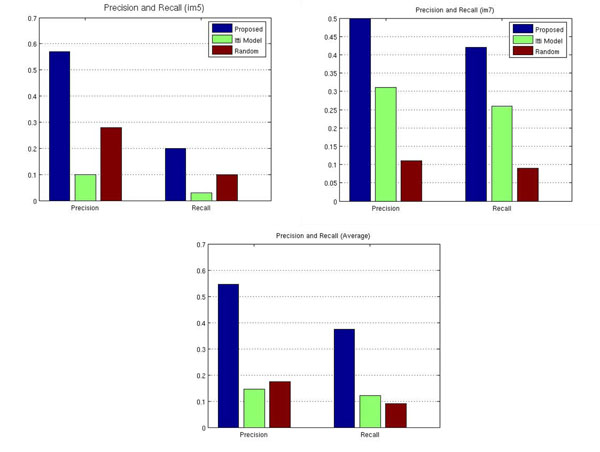
Precision and recall measures for image 5, 7 and average of the dataset.

In figure 4, it is observed the performance for every method for VS 5, 7 and the general average. It is worthy to point out that the Itti’s performance is quite variable regarding the whole set, basically because it is a very local strategy which is unable to capture concepts, as required in this case.

## Discussion

The method presented in this article is based on the selection of examples for extracting diagnostic regions of interest in histopathologic images. Information is integrated from different low-level features, extracted from the VS. The results indicate that introduction of expert knowledge, through a positive (negative) learning, has a positive impact on the identification of these regions. Likewise, experiments demonstrated that visual attention methods, based only on low-level information of the image, are unadequate to solve this problem, probably because the estimated regions with such models are not always related to the visual patterns associated to pathologies. An important conclusion of this work is that the presented method is quite general, it was herein applied to very different types of histological samples and results are very consistent.

The proposed model demonstrated a higher performance regarding precision and recall measurements when comparing with methods based on attentional models, showing that prior knowledge introduction and integration of multiple image features highly improves the RoI identification. Yet there exist method for finding RoIs, it is worthy to strengthen out that most of these methods are designed for a specific pathology. On the contrary, our method is sufficiently general as to be easily trained for any other pathology, using one block as a target example and another as a distractor.

The variables of this model are basically associated to the partition size of the VS, the selection of the example block and the feature integration process. Regarding the former factor, it is better to have a random criterion since it is very arguable if a determined size completely captures main features of a RoI. The selection of the block examples are basically function of the pathologist’s expertise, but in difficult cases our approach could be modified to using a set of block rathern than a single one. Finally, the integration process follows a simple rule, which consists in promoting local signals, this obviously can be improved and addressed to detect more general features. Nevertheless, in the present investigation the method was enough for having good region estimations. Future work includes then further investigation on integration strategies which better follow regional criteria as well as selection of an optimal number of blocks which reach a sparse representation of the original signal, that is to say, blocks which capture the semantic of each region.

## Competing interests

The authors declare that they have no competing interests.
